# Reference values for spirometry – report from the Obstructive Lung Disease in Northern Sweden studies

**DOI:** 10.3402/ecrj.v2.26375

**Published:** 2015-07-20

**Authors:** Helena Backman, Anne Lindberg, Anders Odén, Linda Ekerljung, Linnéa Hedman, Annette Kainu, Anssi Sovijärvi, Bo Lundbäck, Eva Rönmark

**Affiliations:** 1 The OLIN Unit, Division of Occupational and Environmental Medicine, Department of Public Health and Clinical Medicine, Umeå University, Umeå, Sweden; 2 Division of Medicine, Department of Public Health and Clinical Medicine, Umeå University, Umeå, Sweden; 3 Division of Mathematical Sciences, Chalmers University of Technology, Gothenburg, Sweden; 4 Krefting Research Centre, Institute of Medicine, University of Gothenburg, Gothenburg, Sweden; 5 HUCH Heart and Lung Center, Peijas Hospital, Helsinki University Central Hospital, Helsinki, Finland; 6 HUS Medical Imaging Centre, Department of Clinical Physiology and Nuclear Medicine, Helsinki University Central Hospital, Helsinki, Finland

**Keywords:** reference equations, reference sample, Z-scores, percentiles, linear regression, spline functions

## Abstract

**Background:**

Abnormal lung function is commonly identified by comparing observed spirometric values to corresponding reference values. It is recommended that such reference values for spirometry are evaluated and updated frequently. The aim of this study was to estimate new reference values for Swedish adults by fitting a multivariable regression model to a healthy non-smoking general population sample from northern Sweden. Further aims were to evaluate the external validity of the obtained reference values on a contemporary sample from south-western Sweden, and to compare them to the Global Lung Function Initiative (GLI) reference values.

**Method:**

Sex-specific multivariable linear regression models were fitted to the spirometric data of *n*=501 healthy non-smoking adults aged 22–91 years, with age and height as predictors. The models were extended to allow the scatter around the outcome variable to depend on age, and age-dependent spline functions were incorporated into the models to provide a smooth fit over the entire age range. Mean values and lower limits of normal, defined as the lower 5th percentiles, were derived.

**Result:**

This modelling approach resulted in unbiased estimates of the spirometric outcomes, and the obtained estimates were appropriate not only for the northern Sweden sample but also for the south-western Sweden sample. On average, the GLI reference values for forced expiratory volume in one second (FEV_1_) and, in particular, forced expiratory vital capacity (FVC) were lower than both the observed values and the new reference values, but higher for the FEV_1_/FVC ratio.

**Conclusion:**

The evaluation based on the sample of healthy non-smokers from northern Sweden show that the Obstructive Lung Disease in Northern Sweden reference values are valid. Furthermore, the evaluation based on the south-western Sweden sample indicates a high external validity. The comparison with GLI brought further evidence to the consensus that, when available, appropriate local population-specific reference values may be preferred.

Dynamic spirometry is an important tool for the assessment of lung function in daily clinical practice. Abnormal lung function is identified by comparing an observed value with a reference value. There are a large number of published reference values for spirometry available (1–14). The Global Lung Function Initiative (GLI), an European Respiratory Society (ERS) task force, presented new multi-ethnic reference values for spirometry in 2012 (10), which currently are endorsed by several respiratory societies such as the ERS and the American Thoracic Society (ATS) (10, 15). However, it has recently been shown that the GLI reference values may not be appropriate in all countries (16–20).

It is essential that the sample from which the reference values are derived is representative for the contemporary healthy non-smoking population. The age range and other anthropometric, ethnic, environmental, and socio-economic factors must be considered, since such factors can affect lung function (21). In addition, the spirometric measurements should be performed in line with recommended guidelines (22–25). It is also important that reference values for spirometry are evaluated and updated continuously (22, 24, 25).

Linear regression models have commonly been used to model lung function. In most models, the predictors are sex, age, and height, but sometimes also weight and ethnicity (1–6, 9–13). It has been shown that subjects of European ancestry (Caucasians) have larger lung volumes compared with subjects of other races/ethnicities (22, 26, 27). Height is a proxy for chest size, and women have smaller lung volumes than men. Age is a proxy for maturity, and lung volumes increase by age during childhood and adolescence followed by a plateau with a subsequent decrease, with a starting point some years post adolescence (3, 10, 23). In general, there is a high variability in the lung function development in elderly subjects and the age dependence in later stages of life is less studied (24).

Recently, progress has been made in the area of modelling lung function and the Lambda-Mu-Sigma (LMS) method imbedded in the generalised additive models for location, scale and shape (GAMLSS) models (28, 29) is preferred by some authors (12, 30). This method was used to derive the GLI reference values (10, 31). Beside the mean, the GAMLSS allow for skewness and kurtosis to be modelled. It is also common to use spline functions to allow both the predicted estimates and the standard deviation (SD) to vary non-linearly as functions of an explanatory variable (9, 10, 12, 30). Previous studies have shown that not only the predicted mean but also the SD vary with age, especially when including ages from childhood to adulthood (10, 31), while a large but somewhat older study indicated constant variance for adults (32).

The aim of this study was to estimate new up-to-date reference values for spirometry for adults of European ancestry by fitting a multivariable regression model to data from Caucasian healthy non-smokers sampled from the general population of northern Sweden. Further aims were to evaluate the external validity of the new reference values on contemporary data of healthy non-smokers from south-western Sweden and to compare them with the GLI reference values.

## Material and method

### The northern Sweden reference sample

**Fig. 1:**
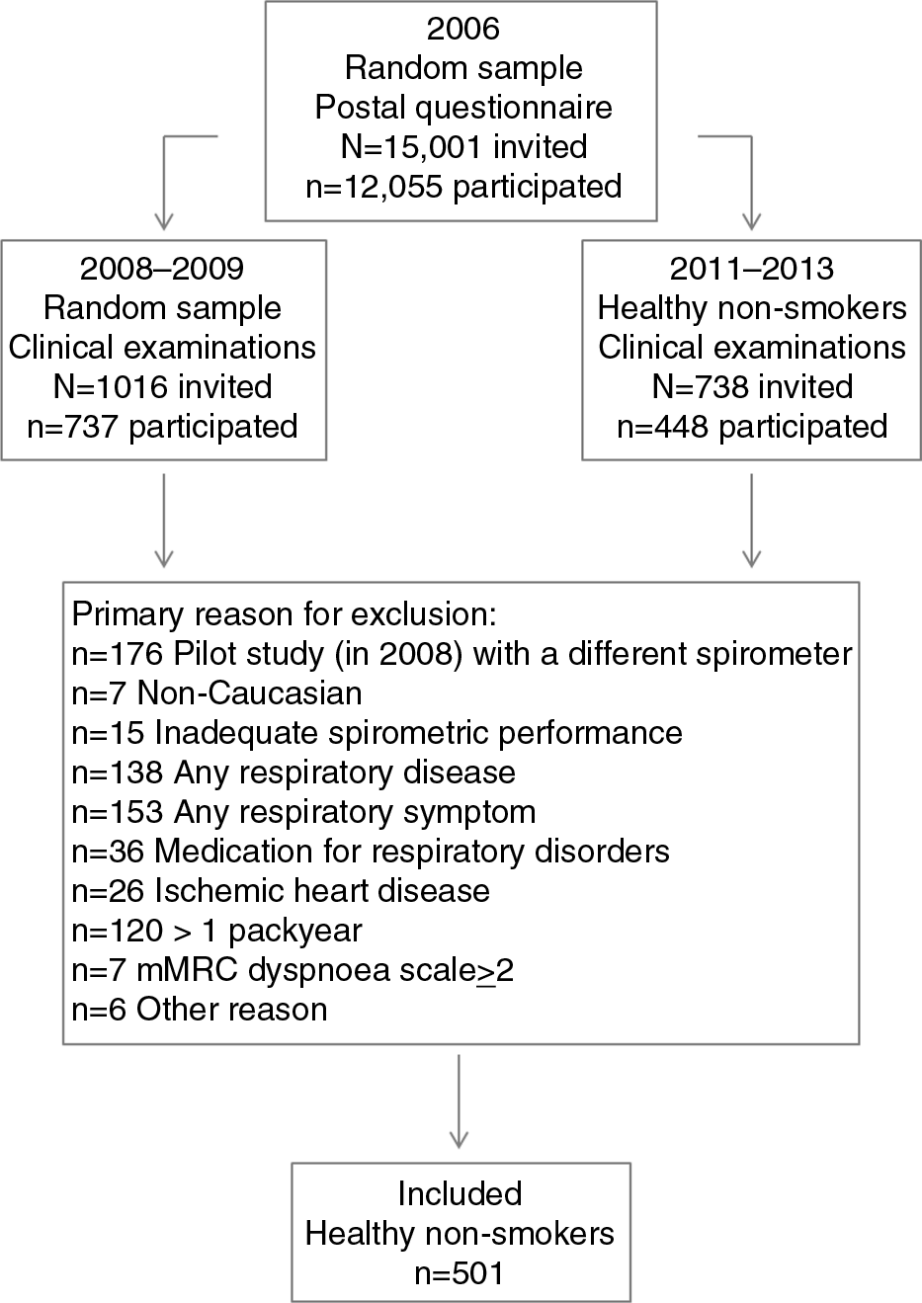
Study flow chart.

As a part of the Obstructive Lung Disease in Northern Sweden (OLIN) studies, 1,016 randomly selected respondents from a large postal questionnaire survey in 2006 (33) were invited to clinical examinations in 2008–2009. Of them, 737 subjects (72.5%) aged 21–86 years participated in structured interviews and spirometry (34). In 2011–2013, 738 additional healthy non-smokers according to the 2006 questionnaire survey were invited to identical examinations, and 448 subjects (60.6%) aged 25–91 years participated (17). The study flow chart is illustrated in Fig. 1. Information about respiratory diseases and symptoms, other diseases, and smoking history was collected at the interview. The study was approved by the Regional Ethical Review Board at Umeå University, Sweden.

Healthy non-smokers were defined as having none of the following criteria: usually wheeze when breathing; sputum production most days in periods of 3 months per year; ever have had asthma; been diagnosed as having asthma, chronic bronchitis, COPD or emphysema by a physician; ever use of asthma medication regularly or when needed; ever use of medication for chronic bronchitis, COPD, or emphysema; ever had ischemic heart disease; wheeze in past 12 months with concurrent breathlessness; wheeze in past 12 months without having a cold; wheeze in the past 12 months most days per week; any other disability that could affect the lung capacity; or mMRC dyspnoea scale≥2. A further exclusion criterion was a cumulative life-long smoking history of>1 pack year.

The age was calculated by one decimal point as the difference between date of birth and date of examination. Date of birth was collected from the Swedish national registry. Height was measured without shoes with an accurate stadiometer with 0.5 cm precision. Weight was measured with 0.5 kg precision with empty pockets without jacket and shoes. Caucasian ethnicity was defined as a subject of European ancestry. Two Jaeger Masterscope spirometers (JLAB version 5.21 software, CareFusion, Würzburg, Germany) were used to measure forced expiratory volume in one second (FEV_1_), forced expiratory vital capacity (FVC), and slow expiratory vital capacity (SVC). The procedure followed the ATS/ERS recommendations (35) but with a reproducibility criterion of≤5% instead of≤150 ml deviation from the second highest value. The same highly experienced, trained, and qualified research nurses performed the measurements throughout the study. The spirometers were calibrated every morning. The highest value for FEV_1_, FVC, and SVC was recorded for each subject and at least three up to a maximum of eight measurements were performed to fulfil the reproducibility criterion. The mean (median) absolute differences between the highest and the second highest values were: 38 ml (27 ml) for women and 59 ml (45 ml) for men in FEV1, and 53 ml (44 ml) for women and 69 ml (54 ml) for men in FVC. In total, 2.8% of the FEV1 measurements and 6.1% of the FVC measurements deviated>150 ml. Vital capacity (VC) was defined as the highest value of FVC and SVC. In total, 501 subjects, 49% women, were identified as healthy non-smoking subjects of European ancestry with adequate spirometry quality. Further details regarding the measurements of lung function can be found in the Appendix.

### Derivation of the OLIN reference values based on the northern Sweden reference sample

Normal Q–Q plots were used to evaluate and confirm normal distribution of the spirometric indices FEV_1_, FVC, SVC, VC, the FEV_1_/FVC ratio and the FEV_1_/VC ratio, and linear regression was used to model each outcome measure. Sex-specific multivariable linear regression models were estimated for each of the spirometric indices, with age and height as independent variables. The relationships with weight were also investigated, but as model improvements were absent or negligible, weight was omitted to keep the models parsimonious. Age-dependent spline functions were incorporated in each model to allow the outcome variable to vary smoothly over the age range. A common assumption of multivariable linear regression is that the SD around the regression function is constant (homogeneous variance). This assumption was extended to include the case when the SD is a linear function of one of the regression variables (an explanatory variable), in this case age. Further details of the statistical modelling are described in the Appendix.

### Model accuracy check

Z-scores (standardised residuals) and percentiles were calculated for each subject. If the observed value has a perfect normal distribution with mean and SD equal to the mean and SD of the reference values, then mean and SD of the percentile point will be 0.50 and 0.2887, respectively. Equivalently, the mean and SD of the Z-score will be 0 and 1, respectively. These facts were used to statistically test the accuracy of the estimations. A p≥0.05 implies good model accuracy. In addition, possible relationships between Z-scores and age, height, weight, and sex were examined by ordinary linear regression models, with significant associations presented as Beta-coefficients. If the reference values are in perfect agreement with the observed values, no such relationship will exist.

### The south-western Sweden sample

To evaluate the external validity of the OLIN reference values, they were also applied to spirometry data of a general population sample from another region in Sweden. In 2009–2012, clinical examinations were performed on a sample aged 17–78 years from the Swedish region of West Gothia (36). The examinations included dynamic spirometry also using a Jaeger Masterscope spirometer (JLAB version 5.03 software, CareFusion) and a structured interview with the same questions as in the interview for the northern Sweden sample. In total, 2,000 subjects were invited to participate in the clinical examinations, of which 1,172 (59%) participated. The study was approved by the Regional Ethical Review Board at the University of Gothenburg. From the participants, 358 healthy non-smoking subjects of European ancestry ≥22 years of age, 54% women, were identified using the same eligibility criteria for healthy non-smokers as in the northern Sweden reference sample. Percent of predicted, Z-scores (standardised residuals) and percentiles based on the OLIN reference values were calculated for each subject.

### Comparison with the GLI reference values

The predicted sex- and age-specific FEV_1_, FVC and FEV_1_/FVC ratio (mean_OLIN_ and LLN_OLIN_) was compared to predictions by the GLI reference values (mean_GLI_ and LLN_GLI_). The comparisons are displayed in graphs, for a male of 180 cm and a female of 165 cm, which are approximately the sex-specific average heights in Sweden.

## Results

### Equations for the OLIN reference values for spirometry

As a result of the modelling technique, the SD, reference value (mean), and lower limit of normal (LLN, defined as the lower 5th percentile) for each of the spirometric indices are calculated by the following formulas:

SD: A+B*age

Mean: (B(J)*X(J))*SD

LLN: Mean −1.645*SD

where possible values for J ranges from 1–5, age is expressed in years, and the coefficients A, B, B1-5 and the variables X1-5 are found in Table 1. Calculation examples are illustrated in the Appendix.

**Table 1:** Equation coefficients for the OLIN reference values for spirometry

		A	B	B1	B2	B3	B4	B5
FEV1	Women	0.3832	−0.0013797	−6.236984	−0.001575	−0.002130	0.000881	0.097457
	Men	0.5335	−0.0013209	−6.792881	−0.016061	−0.000654	−0.000631	0.092415
FVC	Women	0.4835	−0.0009121	−7.504292	−0.006537	−0.001433	−0.000418	0.101606
	Men	0.6515	−0.0009156	−8.145885	−0.024025	−0.000089	−0.000888	0.100738
SVC	Women	0.4890	−0.0004222	−6.585401	−0.018584	−0.000965	−0.000754	0.096274
	Men	0.6842	−0.0013804	−9.466451	−0.013372	−0.000253	−0.000410	0.105695
VC	Women	0.4728	−0.0004556	−7.174368	−0.016404	−0.001117	−0.000775	0.101837
	Men	0.6852	−0.0014865	−9.237482	−0.012298	−0.000215	−0.000550	0.104602
FEV1/FVC	Women	0.0414	0.0003501	21.774779	−0.121986	0.000235	0.002045	−0.014863
	Men	0.0474	0.0000904	20.349431	−0.034677	−0.000816	0.000313	−0.018407
FEV1/VC	Women	0.0397	0.0004102	21.585726	−0.134590	0.000216	0.002355	−0.012948
	Men	0.0510	0.0000706	19.348156	−0.033104	−0.000538	−0.000496	−0.019745

The standard deviation (SD) is calculated by the following formula: A+B*age. The reference value (i.e., the mean) is calculated by the following formula: (Σ B(J)*X(J))*SD. The LLN (lower limit of normal) is calculated by the following formula: Mean-1.645*SD. FEV1=Volume exhaled in the first second of a forced expiratory manoeuvre. FVC=Forced expiratory vital capacity. SVC=Slow expiratory vital capacity. VC=The highest value of FVC and SVC. X1=1. X2=Age (in years). X3=max(min(age-40, 20), 0)^2^+40*max(age-60, 0). X4=max(min(age-60, 20), 0)^2^+40*max(age-80, 0). X5=Height (in cm).

The mean ages for the 244 women and 257 men in the reference sample from northern Sweden were 49.2 (range 22–91) and 46.6 (range 22–86) years, respectively. The mean height was 163.3 cm (range 139.0–181.0) for women and 178.9 cm (range 162.5–198.0) for men, and the mean weight was 68.2 kg (range 45.0–118.0) for women and 84.8 kg (range 56.0–148.0) for men. These are the ranges of age, height, and weight in which the equations for the OLIN reference values are valid.

### Model accuracy check based on the northern Sweden reference sample

Mean observed and predicted values, observed values as percent of predicted values, Z-scores and percentiles for the reference sample (*n*=501) are shown in Table 2, stratified by sex and three age groups. The mean observed values and predicted values were close to identical, which yielded mean percent of predicted close to 100%. Correspondingly, the mean Z-scores were approximately 0 with SDs close to 1, and mean percentile points are approximately 0.5 with SDs close to 0.288. Statistical testing revealed that the models for all spirometric indices were concordant with the observed values. These results were equivalent across all age groups and for both sexes. No significant associations between the Z-scores and sex, age, height or weight were found.

**Table 2:** Evaluation of the OLIN reference values on the northern Sweden reference sample

		Observed values		Predicted values		Predicted LLN		% of predicted		Z-score		Percentile point		
							
Age group		Mean	SD	Mean	SD	Mean	SD	Mean	SD	Mean	SD	Mean	SD	*P*
Among women														
22–39 years *n*=106	FEV1	3.318	0.411	3.316	0.209	2,763	0.205	100.0	10.4	0.006	1.018	0.4919	0.280	0.773
	FVC	4.141	0.539	4.122	0.280	3.378	0.278	100.4	10.9	0.043	1.003	0.5060	0.262	0.831
	SVC	4.157	0.554	4.146	0.280	3.365	0.279	100.2	11.1	0.024	0.983	0.5047	0.264	0.867
	VC	4.195	0.545	4.181	0.284	3.428	0.283	100.3	10.9	0.032	1.001	0.5053	0.266	0.850
	FEV1/FVC	0.804	0.054	0.809	0.006	0.720	0.008	99.5	6.6	−0.069	1.003	0.4809	0.283	0.496
	FEV1/VC	0.793	0.055	0.796	0.006	0.708	0.008	99.6	6.8	−0.051	1.008	0.4891	0.280	0.698
40–59 years *n*=74	FEV1	2.973	0.401	2.975	0.282	2.456	0.271	100.0	10.6	−0.008	0.990	0.5041	0.297	0.903
	FVC	3.781	0.529	3.808	0.338	3.086	0.332	99.5	11.9	−0.062	1.033	0.4776	0.291	0.504
	SVC	3.815	0.576	3.834	0.334	3.064	0.331	99.7	13.2	−0.041	1.084	0.4929	0.289	0.832
	VC	3.852	0.540	3.874	0.338	3.133	0.335	99.6	12.0	−0.048	1.032	0.4829	0.287	0.610
	FEV1/FVC	0.789	0.062	0.782	0.015	0.686	0.018	100.9	7.5	0.119	1.006	0.5422	0.290	0.209
	FEV1/VC	0.774	0.061	0.769	0.017	0.670	0.021	100.7	7.7	0.095	0.987	0.5320	0.292	0.340
60–91 years *n*=64	FEV1	2.036	0.471	2.034	0.366	1.574	0.348	100.0	14.2	0.006	1.006	0.4982	0.294	0.960
	FVC	2.734	0.667	2.737	0.519	2.054	0.507	99.9	15.0	−0.007	0.978	0.4832	0.289	0.642
	SVC	2.817	0.699	2.814	0.528	2.061	0.522	100.0	15.2	0.007	0.947	0.4881	0.281	0.744
	VC	2.830	0.701	2.831	0.538	2.110	0.532	99.9	15.0	−0.003	0.983	0.4828	0.288	0.636
	FEV1/FVC	0.750	0.069	0.749	0.009	0.638	0.008	100.0	9.1	0.004	1.002	0.5078	0.285	0.829
	FEV1/VC	0.726	0.072	0.726	0.009	0.611	0.009	99.9	9.9	−0.007	1.019	0.5030	0.295	0.934
All ages *n*=244	FEV1	2.877	0.673	2.877	0.593	2.358	0.555	100.0	11.5	0.002	1.002	0.4972	0.287	0.880
	FVC	3.663	0.810	3.663	0.680	2.942	0.656	100.0	12.3	−0.002	1.003	0.4914	0.277	0.642
	SVC	3.706	0.811	3.706	0.660	2.935	0.649	100.0	12.9	0.000	1.002	0.4968	0.275	0.863
	VC	3.737	0.808	3.737	0.670	2.996	0.658	100.0	12.3	−0.001	1.002	0.4927	0.277	0.693
	FEV1/FVC	0.785	0.064	0.785	0.026	0.688	0.035	100.1	7.6	0.007	1.003	0.5065	0.286	0.725
	FEV1/VC	0.770	0.067	0.770	0.030	0.671	0.042	100.0	7.9	0.005	1.002	0.5058	0.287	0.754
Among men														
22–39 years *n*=118	FEV1	4.596	0.580	4.579	0.316	3.775	0.313	100.4	10.3	0.036	1.056	0.5065	0.280	0.807
	FVC	5.760	0.760	5.740	0.432	4.719	0.430	100.3	10.6	0.032	0.985	0.5014	0.277	0.958
	SVC	5.876	0.554	5.856	0.461	4.807	0.459	100.3	10.9	0.032	1.004	0.5038	0.280	0.886
	VC	5.896	0.790	5.875	0.455	4.831	0.453	100.4	10.7	0.034	1.000	0.4967	0.280	0.901
	FEV1/FVC	0.800	0.052	0.800	0.006	0.717	0.007	100.1	6.5	0.011	1.027	0.5043	0.288	0.872
	FEV1/VC	0.782	0.056	0.783	0.008	0.695	0.008	100.0	7.2	−0.007	1.056	0.5001	0.290	0.997
40–59 years *n*=84	FEV1	4.177	0.580	4.193	0.346	3.419	0.338	99.7	11.8	−0.033	1.044	0.4931	0.302	0.827
	FVC	5.283	0.741	5.301	0.448	4.301	0.443	99.8	12.2	−0.028	1.042	0.4972	0.304	0.929
	SVC	5.413	0.744	5.429	0.471	4.412	0.464	99.8	11.7	−0.025	1.013	0.4974	0.301	0.934
	VC	5.428	0.739	5.448	0.464	4.438	0.457	99.7	11.7	−0.031	1.022	0.4967	0.303	0.917
	FEV1/FVC	0.793	0.050	0.792	0.006	0.707	0.009	100.0	6.1	0.009	0.937	0.5075	0.280	0.812
	FEV1/VC	0.771	0.050	0.771	0.010	0.682	0.010	100.0	6.1	0.002	1.086	0.5056	0.276	0.859
60–86 years *n*=55	FEV1	3.285	0.657	3.297	0.503	2.576	0.488	99.7	13.4	−0.025	1.020	0.4899	0.293	0.795
	FVC	4.362	0.838	4.382	0.625	3.419	0.615	99.6	13.6	−0.034	0.991	0.4839	0.285	0.679
	SVC	4.517	0.844	4.535	0.605	3.573	0.590	99.6	13.3	−0.031	0.997	0.4836	0.282	0.674
	VC	4.536	0.837	4.553	0.605	3.603	0.589	99.6	13.0	−0.030	0.992	0.4832	0.280	0.666
	FEV1/FVC	0.754	0.058	0.753	0.014	0.665	0.015	100.2	7.6	0.022	1.065	0.5167	0.284	0.668
	FEV1/VC	0.725	0.064	0.725	0.021	0.633	0.022	100.0	8.5	0.002	1.086	0.5126	0.297	0.746
All ages *n*=257	FEV1	4.179	0.777	4.179	0.616	3.402	0.586	100.0	11.5	0.000	1.002	0.4986	0.289	0.938
	FVC	5.305	0.936	5.306	0.709	4.304	0.690	100.0	11.8	−0.001	1.002	0.4963	0.287	0.837
	SVC	5.434	0.943	5.434	0.708	4.414	0.680	100.0	11.7	0.000	1.002	0.4974	0.286	0.885
	VC	5.452	0.939	5.453	0.705	4.439	0.675	100.0	11.6	−0.001	1.002	0.4970	0.286	0.868
	FEV1/FVC	0.788	0.056	0.787	0.020	0.702	0.022	100.1	6.6	0.013	1.003	0.5080	0.284	0.657
	FEV1/VC	0.766	0.060	0.766	0.025	0.677	0.027	100.0	7.2	−0.002	1.002	0.5046	0.286	0.798

A *P*<0.05 indicates a non-perfect model fit.

### Applying the OLIN reference values to the south-western Sweden sample

The OLIN reference values were applied to the south-western Sweden sample, and percent of predicted, Z-scores and percentiles were calculated for each subject. The mean percent of predicted was close to 100% and mean absolute values of the Z-scores are approximately≤0.4, corresponding to approximately≤3% of predicted, for all spirometric indices in both age groups and for both sexes in the south-western Sweden sample (*n*=358). However, statistical testing revealed that the model fit was not perfect for some of the spirometric indices, especially among women (Table 3). Also, small but statistically significant associations between the Z-scores and sex were found for FEV_1_, SVC, and VC such that women on average had higher Z-scores than men when corrected for age and height (B-coefficients between 0.331 and 0.453). When also corrected for weight, the association between the Z-scores for FVC and FEV_1_/FVC reached statistical significance for female sex (B-coefficients of 0.330 and 0.177, respectively). No other statistically significant associations between the Z-scores and age, height, or weight were found when weight was included, except for the Z-score for FVC where height measured in cm yielded a B-coefficient of 0.018 with p-value 0.049.

**Table 3:** Evaluation of the OLIN reference values on the south-western Sweden sample

		Women							Men						
			
		% of predicted		Z-score		Percentile point			% of predicted		Z-score		Percentile point		
							
Age group		Mean	SD	Mean	SD	Mean	SD	*P*	Mean	SD	Mean	SD	Mean	SD	*P*
22–39 years	FEV1	101.7	10.5	0.176	1.032	0.538	0.279	0.278	98.4	10.9	−0.151	1.038	0.475	0.294	0.466
*n*=68 women	FVC	99.7	10.6	−0.017	0.964	0.482	0.283	0.607	98.6	10.7	−0.135	1.011	0.469	0.301	0.366
*n*=71 men	SVC	99.0	10.8	−0.076	0.952	0.464	0.280	0.304	97.1	10.5	−0.276	0.979	0.425	0.282	0.029
	VC	99.2	10.5	−0.059	0.965	0.467	0.281	0.346	97.3	10.5	−0.254	0.991	0.435	0.287	0.058
	FEV1/FVC	102.1	7.3	0.339	1.156	0.584	0.311	0.016	99.8	7.0	−0.027	0.931	0.498	0.302	0.953
	FEV1/VC	102.6	7.5	0.413	1.163	0.606	0.308	0.002	100.9	7.2	0.132	1.065	0.545	0.285	0.189
40–77 years	FEV1	99.6	11.6	−0.029	1.039	0.489	0.304	0.679	97.5	11.7	−0.205	1.024	0.454	0.281	0.109
*n*=118 women	FVC	96.2	11.1	−0.289	0.926	0.414	0.273	0.001	98.6	12.1	−0.106	1.054	0.468	0.305	0.265
*n*=101 men	SVC	96.4	11.2	−0.256	0.893	0.418	0.265	0.002	97.8	12.1	−0.174	1.064	0.450	0.305	0.082
	VC	96.2	10.9	−0.287	0.911	0.411	0.266	0.001	98.2	11.8	−0.145	1.062	0.435	0.304	0.024
	FEV1/FVC	103.3	7.8	0.405	0.963	0.616	0.266	<0.001	99.0	6.3	−0.155	0.931	0.458	0.275	0.144
	FEV1/VC	103.3	8.3	0.375	0.978	0.616	0.286	<0.001	99.4	7.0	-0.088	0.954	0.480	0.279	0.486
All ages	FEV1	100.4	11.2	0.046	1.038	0.507	0.295	0.741	97.9	11.4	−0.183	1.027	0.463	0.286	0.093
*n*=186 women	FVC	97.5	11.0	−0.189	0.947	0.439	0.278	0.004	98.6	11.5	−0.118	1.034	0.469	0.302	0.159
*n*=172 men	SVC	97.3	11.1	−0.176	0.920	0.435	0.271	0.002	97.5	11.4	−0.217	1.027	0.439	0.295	0.006
	VC	97.3	10.8	−0.204	0.935	0.432	0.272	0.001	97.8	11.3	−0.190	1.032	0.448	0.297	0.018
	FEV1/FVC	102.9	7.6	0.381	1.035	0.604	0.283	<0.001	99.3	6.6	−0.102	1.013	0.475	0.286	0.256
	FEV1/VC	103.0	8.0	0.389	1.046	0.612	0.293	<0.001	100.0	7.1	0.003	1.004	0.506	0.283	0.785

A *P*<0.05 indicates a non-perfect model fit.

### Comparison with GLI

The predicted mean_GLI_ for FEV_1_ in the northern Sweden reference sample was on average 67 ml lower (*p*<0.001) than the predicted mean_OLIN_ for women and 103 ml lower (*p*<0.001) than the predicted mean_OLIN_ for men. For FVC, the predicted mean_GLI_ was on average 203 ml lower for women (*p*<0.001) and 190 ml lower for men (*p*<0.001). The average predicted mean_GLI_ for the FEV_1_/FVC ratio was 0.028 units higher (*p*<0.001) for women and 0.011 units higher for men (*p*<0.001).

**Fig. 2:**
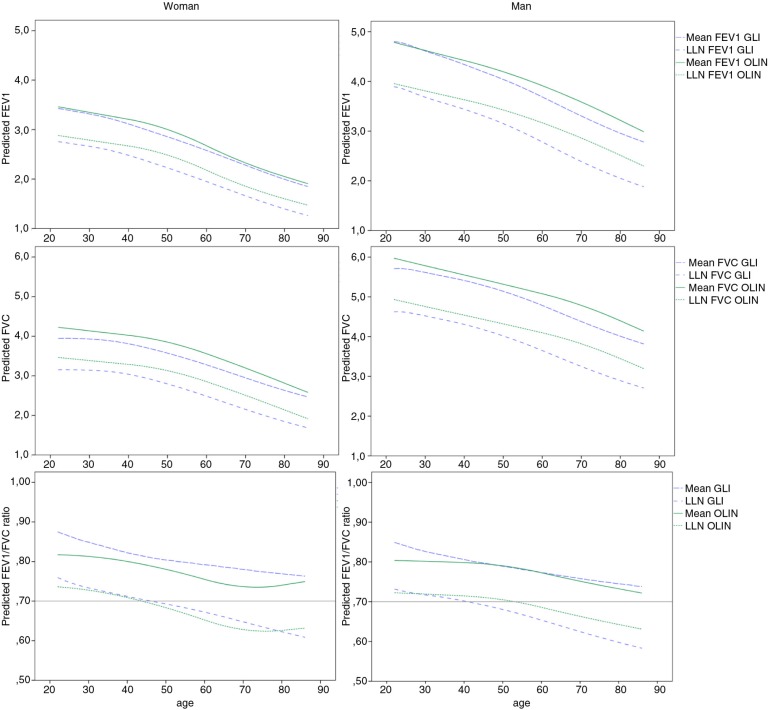
(a–f) Sex- and age-dependent decline in the OLIN reference values (mean and LLN) in comparison with GLI. The predicted mean and LLN for the OLIN and the GLI reference values are plotted against age, for FEV_1_, FVC and the FEV_1_/FVC ratio, respectively. Figures (a), (b) and (c) display predicted values for a 165 cm tall woman, while (d), (e) and (f) display predicted values for a 180 cm tall man.

To make further comparisons to the GLI reference values, the predicted reference values of FEV_1_, FVC and the FEV_1_/FVC ratio (mean and LLN) for a woman and man of average height are plotted by age in Fig. 2a–f. The comparisons showed that for a man of average height, mean_GLI_ for both FEV_1_ and, in particular, for FVC, were lower than mean_OLIN_ across the ages 22–86 years. For a woman of average height, mean_OLIN_ and mean_GLI_ for FEV_1_ were similar, but mean_GLI_ FVC is constantly lower than mean_OLIN_ FVC. Consequently, the mean_GLI_ FEV_1_/FVC ratio was also consistently lower than the mean_OLIN_ for the woman. For a woman of average height, the LLN_GLI_ and LLN_OLIN_ for the FEV_1_/FVC ratio was well in concordance throughout the age span, but less so for FEV_1_ and FVC where LLN_GLI_ was consistently lower than LLN_OLIN_. The pattern was somewhat different for the average height man, where the gap between LLN_GLI_ and LLN_OLIN_ increased with increasing age for all three spirometric indices. LLN_GLI_ for the FEV_1_/FVC ratio reached below 0.7 at the age between 46 and 47 years for a woman of average height and at the age 39–40 years for a man of average height. The corresponding ages when LLN_OLIN_ reached below 0.7 for the FEV_1_/FVC ratio are 43–44 years for the woman and 53–54 years for the man. Both the LLN_GLI_ and LLN_OLIN_ for FEV_1_/FVC reached below 0.7 in earlier stages in life for taller subjects compared to for shorter subjects of both sexes.

## Discussion

The modelling approach in this study produced unbiased estimates of mean observed values in the reference sample from northern Sweden. The approach to model lung function by linear regression is a powerful technique, which we successfully extended to let the SD depend on age. In general, the GLI reference values for FEV_1_ and FVC were lower than the corresponding values in our sample, but higher for the FEV_1_/FVC ratio. In addition, the SD for the GLI reference values was larger for all spirometric indices, which can impact the LLN. Furthermore, the results indicate a high external validity for Swedish conditions, when evaluated on the sample from south-western Sweden.

Historically, multivariable linear regression has commonly been used to model spirometric outcomes since it is robust and straight-forward. However, linear regression assumes that the outcome variable is normally distributed; an assumption not always true for all spirometric indices (3, 10). In addition, traditional multivariable linear regression modelling does not incorporate the dependence that exists between the dispersion around the spirometric outcome and age (10, 25), that is, one of the independent or explanatory variables. In this study however, we found all spirometric outcomes to be reasonably normally distributed. A new approach to model spirometric outcomes by linear regression is presented, where the sex-specific spirometric outcomes are predicted by age and height, and where the SD also is allowed to linearly depend on age. Because of the strengths of linear regression modelling mentioned above, this approach presents a useful technique which can be used to model lung function in an efficient way.

It is essential that reference values are evaluated and updated frequently in order to confirm that the classifications of normal and abnormal reflects the realities of the contemporary general population (22, 24, 25). It has recently been shown that the GLI reference values may not be appropriate for all countries (16–20). The comparison between these new Swedish reference values, that is, the OLIN reference values, and the GLI reference values show that, on average, both the predicted mean values and LLN for FEV_1_, FVC and FEV_1_/FVC for a woman and man of average height differ across the entire life span with differences generally more pronounced in older ages. Despite recent debate of its appropriateness (37), classification of airway and lung disease severity often relies on FEV_1_ and FVC expressed as percent of the predicted value (38). Also, since the LLN for the FEV_1_/FVC ratio is used as a key element for the diagnosis of airway obstruction, the use of GLI may lead to invalid diagnosis of airway obstruction along with erroneous classification of airway and lung disease severity in the Swedish population (17).

A prominent advantage of the GLI reference values is that they cover an extensive age span and different ethnicities, and thus provide estimates without junction points. The GLI reference values for Caucasians are based on collated data from>57,000 subjects, and estimations based on such an extensive amount of data are likely to be valid. Undoubtedly, the GLI is an extremely valuable contribution to the field of lung function reference values, and provide the opportunity to compare a subject or a population to the world-wide average within each ethnicity which is of substantial value for, for example, international multi-centre studies. Quanjer et al. (39) has previously shown that the GLI SDs are expected to be larger compared to SDs derived from smaller populations due to the large number of data subsets included in the estimation of GLI, an expectation which is confirmed in our sample (17). Quanjer et al. argue that although the LLN according to GLI will identify a somewhat larger proportion of subjects as below LLN in smaller populations compared to in larger populations, this will not lead to any bias since the smaller populations merely are subsets of the population at large. However, we argue that since there are considerable differences in, for example, environmental pollution, occupational exposures and socio-economic factors which can affect lung function between regions (21), bias can indeed be introduced and observed differences should not be neglected. In addition, although the GLI reference values currently are recommended by many respiratory societies, the consensus is still that, when available, appropriate and applicable local population-specific reference values may be preferred (22, 23, 25). This consensus is confirmed in our study.

An advantage of our study is that, along with FEV_1_ and FVC, reference values are provided also for SVC and for the highest value of the forced and slow VC, that is, the VC. Although the FVC is a recognised proxy for the VC, additional information of the lung function can be obtained by measuring the SVC, especially in subjects where FVC is reduced by air-trapping due to small-airways obstruction, for example, at early stages of COPD. For example, it is common practice that the FEV_1_/VC ratio is relied on when diagnosing obstruction and the GOLD criteria for the diagnosis of COPD recognise this alternative to the FEV_1_/FVC ratio (38), but hitherto few recently published studies provide reference values (and LLN) also for SVC and VC. A weakness of our study is that the age range does not cover children or adults younger than 22 years, that sitting height was not recorded although it has been argued to be a better predictor than standing height (20, 26) and that the geographical coverage was limited to northern Sweden. The small but still observable differences in lung function between the samples from northern and south-western Sweden could possibly be due to minor technical or procedural differences, which are not uncommon (39), while substantial biological differences are more unlikely.

Two spirometers, instead of only one, were used for the northern Sweden sample. However, they were of identical brand and model which limits the possible bias. The spirometers were calibrated on a daily basis, but the syringes were not sent for yearly calibration according to the ERS/ATS recommendations and there is a lack of calibration traceability. Further, future studies would most likely benefit from European Spirometry Driving Licenses for the staff and deep insight in previous study protocols such as the NHANES (40, 41). However, all measurements in this study were performed by the same three well-trained research nurses with an extensive experience. Also, seasonal variation effects on lung function are avoided due to the random sampling over several years, regardless of season.

The random sample size of 501 subjects is greater than the sample sizes in commonly used Swedish reference values (1, 4, 5) and suffices to produce valid estimates, results in line with previous studies (39). It is possible that our eligibility criteria are too strict and consequently exclude too many subjects, particularly among the elderly. However, obstructive lung diseases, smoking history, breathlessness, cough and wheeze have been shown to be feasible exclusion criteria to define healthy non-smokers in reference populations for spirometry (25, 42). Since the same structured interview questions were used in northern and south-western Sweden, these criteria could be applied identically for both samples. The reference sample from northern Sweden constitutes a representative sample of the general healthy non-smoking population of the area. Thus, bias introduced by including smokers (1, 4, 5) or by using selected samples such as workers from specific industries (13) or respiratory healthy patients from certain hospitals is avoided. The evaluation of the reference values in the south-western Sweden sample yielded mean Z-scores close to 0 and Z-score SDs close to 1, with corresponding mean percent of predicted close to 100%, for all six different spirometric parameters. Although statistical differences were observed for some of the spirometric parameters, the evaluations reveal a high external validity.

To summarise, the evaluation of the OLIN reference values based on the reference sample of healthy non-smokers from northern Sweden show that the models are valid. Further, the evaluation based on the healthy non-smokers from south-western Sweden indicates a high external validity of the model. The comparison with GLI brought further evidence to the consensus that, when available, appropriate local population-specific reference values may be preferred.

## Appendix

Sex-specific normal Q–Q plots of observed values for the six spirometric indices in the northern Sweden reference sample

### Details of the statistical modelling

For each individual we consider a vector **x** of independent variables and x is a component of the vector. The SD σ(x) is assumed to be

σ(x)=a+b⋅x,

where a and b are parameters to be estimated together with a set of parameters describing the mean µ of the dependent variable as a function of the components of the vector **x**. The log-likelihood function is a sum of terms

1 $$-\text{0}.5\cdot \text{log}(2\cdot \pi )-\text{log}(\sigma (\text{x}))-0.5\cdot {\left(\text{y}-\mu (\text{x})\right)}^{2}/\sigma^{\text{2}}(\text{x})$$

The mean µ(**x**) does not need to be linear as a function of the components of x, but the mean has to be linear as a function of the parameters.

The variable Y/σ(x) has a normal distribution and the regression is homogeneous. By use of an iterative procedure, where a provisional function approximating σ(x) is used, the parameters of µ(**x**) are approximately solved. Given the provisional parameters of µ(**x**), new values of a and b approximating σ(x) will be found by solving a linear system of equations (two equations). The system of equations is found by derivation of the log-likelihood function, putting the derivatives equal to zero and making Taylor expansions (linearize) of the equations. New provisional values of a and b are then obtained and Y/σ(x) is again approximately calculated, etc. Finally, repeated solutions of all the parameters give the same results. An efficient way of presenting (writing) the derivatives of the log-likelihood function simplifies programming and procedure checking.

The derivative of (1) with respect to a, here denoted FA1, is

$$\text{FA1}=-1/(\text{a}+\text{b}\cdot \text{x})-0.5\cdot {\left(\text{y}-\mu (\text{x})\right)}^{2}\cdot (-2)/(\text{a}+\text{b}\cdot \text{x}{)}^{3}$$

The second derivative d^2^/da^2^ denoted FA2, which we need for the Taylor expansion, is

$$\text{FA2}=1/(\text{a}+\text{b}\cdot \text{x}{)}^{2}-0.5\cdot {\left(\text{y}-\mu (\text{x})\right)}^{2}\cdot (-2)\cdot (-3)/(\text{a}+\text{b}\cdot \text{x}{)}^{4}$$

and d^2^/dadb, which we denote FAB, is

$$\text{FAB}=\text{x}/(\text{a}+\text{b}\cdot \text{x}{)}^{2}-0.5\cdot {\left(\text{y}-\mu (\text{x})\right)}^{2}\cdot (-2)\cdot (-3)\cdot \text{x}/(\text{a}+\text{b}\cdot \text{x}{)}^{4}$$

The derivative of (1) with respect to b, denoted FB1, is

$$\text{FB1}=-\text{x}/(\text{a}+\text{b}\cdot \text{x})-0.5\cdot {\left(\text{y}-\mu (\text{x})\right)}^{2}\cdot (-2)\cdot \text{x}/(\text{a}+\text{b}\cdot \text{x}{)}^{3}$$

The second derivative d^2^/db^2^, denoted FB2, is

$$\text{FB2}={\text{x}}^{\text{2}}/(\text{a}+\text{b}\cdot \text{x}{)}^{2}-0.5\cdot {\left(\text{y}-\mu (\text{x})\right)}^{2}\cdot (-2)\cdot (-3)\cdot {\text{x}}^{\text{2}}/(\text{a}+\text{b}\cdot \text{x}{)}^{4}$$

The derivative d^2^/dbda, denoted FBA, equals d^2^/dadb, denoted FAB, which is given above. Thus

$$\begin{array}{c} \text{FB1}=\text{FA1}\cdot \text{x}\\ \text{FB2}=\text{FA2}\cdot {\text{x}}^{\text{2}}\\ \text{FBA}=\text{FAB}=\text{FA2}\cdot \text{x} \end{array}$$

### Further evaluation of the statistical models

Predicted values based on mean heights for the *n*=501 subjects in the reference sample are plotted against age in the graphs below in order to further evaluate that the sample size is sufficient and that the models are valid. Height is predicted by sex and age in these graphs. Mean±1.645*sd for each of the spirometric indices are presented.

### Details regarding measurements of lung function

Age was calculated with one decimal point by subtracting the date of birth from the examination date. Date of birth was collected from the Swedish national registry.

Measurements of stature (height) were carried out by stadiometers available at the different healthcare centres in the county of Norrbotten where the clinical examinations took place. All measurements were recorded with the head positioned in ‘the Frankfurt plane’ with 0.5 centimetre intervals. The measurements were performed without shoes on but all subjects were allowed to keep their in-door socks on during measurements.

The barometric pressure was collected from the SMHI (the Swedish Meteorological and Hydrological Institute) website which publishes the current barometric pressure for most towns in Norrbotten County on a daily basis www.smhi.se/vadret/vadret-i-sverige/observationer#. Room temperature was measured with a thermometer by the examining nurse at the time of calibration. The assumed temperature in the pneumotach during expiration was 37 degrees Celsius.

All equipment including the two calibration syringes used in this study were newly purchased, serviced and calibrated at study start. The service providers offered support during the study. The syringe was placed on a table during calibration; no ‘hands on’ was applied. The calibration was performed with both the elbow connection and the filter attached. The three flow protocol was applied at study start and at study stop and the results showed no deviance from linearity. The Masterscreens warnings of insufficient forced expiration were enabled. The Lilly screen was ocularly inspected and cleansed according to the recommendations on a daily basis and replaced when necessary. A nose clip was applied during spirometry.

The following filter was used throughout the study:

CareFusion V-892380 MicroGard^®^ IIB, bacteria/viral filter

With both inspiratory and expiratory resistance:

$$<0.04\text{ kPa}/\text{LPSat1L}/\text{sec}(<0.4\text{cm}{\text{H}}_{\text{2}}\text{O}/\text{L}/\text{secat1L}/\text{sec})$$

The research staff served as biological controls continuously during the data collection to check for inconsistencies in spirometer performance, however not in a structured form. No obvious deviations were observed.

### Calculation of OLIN reference values – worked examples

The sex-specific coefficients A, B, B1, B2, B3, B4 and B5 for each of the different spirometric indices are found in Table 1 in the main manuscript.

The formula for calculation of the predicted mean value is as follows:

Mean=(∑B(J)*X(J))*SD

where J ranges from 1 to 5 and SD is calculated as A+B* age)

This can be expressed as follows:

Mean=(X1*B1+X2*B2+X3*B3+X4*B4+X5*B5)*(A+B*age)

The formula for calculation of the lower limit of normal (LLN) is as follows:

LLN=Mean-1,645*SD

This can be expressed as follows:

LLN=Mean-1,645*(A+B*age)

*Example 1 – FEV1 for a 75 year-old woman of 155 cm*

First, X1, X2, X3, X4 and X5 must be calculated:

X1=1

X2=Age in years=75

$$\text{X}3=\text{max}{\left(\text{min}(75-40,20),0\right)}^{2}+40*\text{max}(75-60,0)=\text{max}{\left(20,0\right)}^{2}+40*\text{max}(15,0)=400+600=1000$$

$$\text{X}4=\text{max}{\left(\text{min}(75-60,20),0\right)}^{2}+40*\text{max}(75-80,0)=\text{max}{\left(15,0\right)}^{2}+40*\text{max}(-5,0)=225$$

X5=Height in cm=155

For FEV_1_, the coefficients A, B, B1, B2, B3, B4 and B5 are:

$$\begin{array}{ccc} \text{A} & = & 0,3832\\ \text{B} & = & -0,0013797\\ \text{B}1 & = & -6,236984\\ \text{B}2 & = & -0,001575\\ \text{B3} & = & -0,002130\\ \text{B4} & = & 0,000881\\ \text{B5} & = & 0,097457 \end{array}$$

Mean predicted FEV_1_ and LLN for FEV_1_ is calculated as follows:

$$\begin{array}{c} \text{FE}{\text{V}}_{\text{1}}\text{ mean}=(-6,236984*1-0,001575*75-0,002130*1000+0,000881*225+0,097457*155)*(0,3832-0,0013797*75)=6,818951*0,2797225=1,91\\ \text{FE}{\text{V}}_{1}\text{ LLN}=\text{Mean}-1,645*(0,3832-0,0013797*75)=\text{1},\text{45} \end{array}$$

*
Example 2 – FEV1 for a 45 year-old man of 185 cm*

First, X1, X2, X3, X4 and X5 need to be calculated:

X1=1

X2=Age in years=45

$$\text{X}3=\text{max}{\left(\text{min}(45-40,20),0\right)}^{2}+40*\text{max}(45-60,0)=\text{max}{\left(5,0\right)}^{2}+40*\text{max}(-15,0)=25+0=25$$

$$\text{X}4=\text{max}{\left(\text{min}(45-60,20),0\right)}^{2}+40*\text{max}(45-80,0)=\text{max}{\left(-15,0\right)}^{2}+40*\text{max}(-35,0)=0+0=0$$

X5=Height in cm=185

For FEV_1_, the coefficients A, B, B1, B2, B3, B4 and B5 are:

$$\begin{array}{ccc} \text{A} & = & 0,5335\\ \text{B} & = & -0,0013209\\ \text{B1} & = & -6,792881 \end{array}$$

$$\begin{array}{ccc} \text{B2} & = & -0,016061\\ \text{B3} & = & -0,000654\\ \text{B4} & = & -0,000631\\ \text{B5} & = & 0,092415 \end{array}$$

Mean predicted FEV_1_ and LLN for FEV_1_ is calculated as follows:

$$\text{FE}{\text{V}}_{\text{1}}\text{ mean}=(-6,792881*1-0,016061*45-0,000654*25-0,000631*0+0,092415*185)*(0,5335-0,0013209*45)=9,564799*0,4740595=4,53$$

$$\text{FE}{\text{V}}_{\text{1}}\text{ LLN}=\text{Mean}-1,645*(0,5335-0,0013209*45)=3,75$$
